# Examining the Relationship Between Balance and Functional Status in the Geriatric Population

**DOI:** 10.3390/medsci13030110

**Published:** 2025-08-02

**Authors:** Eleni Vermisso, Effrosyni Stamou, Garyfallia Tsichli, Ioanna Foteinou, Anna Christakou

**Affiliations:** 1 Department of Physiotherapy, School of Health Sciences, University of Peloponnese, 23100 Sparta, Greece; elenivermisso@gmail.com (E.V.); stam.froso@gmail.com (E.S.); garyfalliatsichli@gmail.com (G.T.); photeinouioanna@gmail.com (I.F.); 2 Laboratory of Biomechanics, Department of Physiotherapy, School of Health Sciences, University of Peloponnese, 23100 Sparta, Greece

**Keywords:** assessment, balance, functional status, geriatric population, physiotherapy

## Abstract

Background/Objectives: Aging is associated with a gradual decline in physical capabilities, often leading to impaired balance and reduced functional status, which are major contributors to falls in older adults. Although many studies have assessed these variables independently, a limited amount of research has explored the direct relationship between balance and functional status in a healthy geriatric population. The aim of this study was to investigate the relationship between balance and functional capacity and to assess the influence of demographic factors such as age, comorbidities, smoking status, and history of falls. Methods: A sample of community-dwelling older adults (19 women, 16 men) (*n* = 35), aged 60 years and above (M = 78 years; SD = 9.23) from Sparta, Greece, took part in the present study. Participants were assessed using three validated tools: (a) the Five Times Sit-to-Stand test, (b) the Timed Up-and-Go test, and (c) the Berg Balance Scale. Spearman’s rank correlation coefficient was used for statistical analysis (α = 0.05). Results: Age was positively correlated with poorer performance in the Five Times Sit-to-Stand (r = 0.40; *p* < 0.01) and the Timed Up-and-Go test (r = 0.47; *p* < 0.01) and negatively correlated with Berg Balance Scale scores (r = −0.51; *p* < 0.01). Comorbidities and smoking were also associated with the Berg Balance Scale. A strong negative correlation was observed between balance and the other two functional tests (Five Times Sit-to-Stand: r = −0.51; Timed Up-and-Go: r = −0.66; both *p* < 0.01). Conclusions: The findings highlight the importance of evaluating both balance and functional capacity in older adults as interrelated factors that can significantly influence quality of life and fall risk. Future research with larger and more diverse populations is recommended to confirm the present findings and to use exercise programs to prevent falls in the geriatric population.

## 1. Introduction

The improvement in the quality of life has significantly increased life expectancy [1]. Among older adults, goals such as maintaining social independence, functional mobility, and cognitive abilities are becoming increasingly important and demanding. Functional decline is often the initial symptom of illness in elderly individuals. Such impairments can substantially affect the quality of life and will have a significant impact on future care needs [2]. As individuals age, there is a progressive deterioration in physical abilities, which may hinder the ability to perform fundamental activities of daily living [3]. One of the most prominent and pressing issues in geriatric care is the incidence of falls. Falls constitute a leading cause of injury-related morbidity and mortality in this population, with epidemiological data indicating that one in three individuals over 65 years—and one in two over 80 years—will experience a fall annually [4]. The decline in functional status may be attributed to balance deficits, which lead to reduced quality of life [5]. These deficits contribute to increased risk of inactivity and decreased ability to perform activities of daily living, which are essential for self-care and independence [6]. Studies have indicated that one of the biggest problems after the experience of falls is the fear of falling, which can lead to fall-related injuries, as well as decreased walking speed, balance performance, and physical function [7]. These findings are independent predictors of self-perceived function in older adults. Another important consequence of falls are restrictions on activities, declining health, and increased risks of institutionalization, fractures, and death. These consequences lead to physical and psychological damage and increase the costs of healthcare for the elderly population [8].

Regardless of the presence of comorbidities, advancing age is associated with decreased mobility and progressive disability in the execution of everyday tasks [9]. In the last decade, the percentage of non-independent older adults has reached 21% [10]. Functional capacity is typically assessed through a range of clinical tests, validated questionnaires, and standardized scales, administered under the guidance of physiotherapy professionals. Despite the extensive literature concerning the assessment of functional status and balance capacity in older adults with compromised health, there is a notable lack of studies focusing exclusively on healthy elderly populations, especially in relation to the risk of falls. There is a critical need for the present study, because most existing research on balance and functional status in older adults focuses on individuals with underlying health conditions, such as stroke or neurological impairments. As a result, the current evidence does not accurately reflect the abilities or challenges faced by healthy older adults living in the community. Specifically, Park et al. [11] examined a sample of older individuals who had suffered a cerebrovascular accident (CVA) as a comorbidity, aiming to evaluate improvements in trunk control, balance, and performance of daily activities. In addition, Alenazi et al. [12] investigated the relationship between balance, functional ability, and the number of falls in individuals post-stroke as a comorbidity. Both of these studies examined the relationship between falls and their respective population.

Only a few studies have specifically focused on healthy elderly people. The purpose of the present study is to evaluate the functional status and balance capacity of the healthy geriatric population in Sparta. In contrast to the majority of existing research, which primarily focuses on clinical populations with neurological or musculoskeletal conditions, this study targets a non-pathological aging cohort within the Greek population, which limits the generalizability of international findings to this specific demographic. The findings may inform timely physiotherapeutic interventions, contribute to fall prevention strategies, and support the promotion of autonomy and quality of life in the aging population. As such, the study holds both clinical and public health relevance.

## 2. Materials and Methods

### 2.1. Study Design

This study was approved by the Ethics Committee of the School of Health Studies of the University of Peloponnese (106/03-01-2025); it was a cross-sectional study and in agreement with the declaration of Helsinki’s ethical principles.

### 2.2. Participants

A convenience sample of 35 elderly people volunteered for this explanatory observational study. An a priori power analysis was conducted using G*Power version 3.1.9.7., which showed that at least a sample of 28 participants is necessary in a correlation–bivariate normal model with an 80% power for detecting a large effect and significance criterion of α = 0.05. The sample was taken from the nursing home and the community center for the elderly population of the Municipality of Sparta. Due to its small size, it is recognized to have a risk of type II error in statistics. We recognized the limited applicability and the low replicability of the convenience method of sampling.

Inclusion criteria were as follows: (1) individuals aged over 60 years; (2) participants must be ambulatory. The exclusion criteria were as follows: (1) any neurological, musculoskeletal, or mental problem; (2) injury or surgery in the past 6 months (Table 1).

### 2.3. Instruments

The instruments and clinical tests that were used were valid and reliable:

a. Five Times Sit-to-Stand test (FTSTS) [13].

This test assesses lower limb function. Participants sit on a chair with their arms on their chest, and as quickly as they can, they have to stand up and sit down 5 times. A stopwatch is used to accurately measure the subjects. When the time equals or exceeds 16.7 s, it indicates inadequate, adequate (13.7–16.6), good (11.2–13.6), and very good (<11.2) lower limb strength performance [5]. This test has been shown to be reliable (ICC = 0.93) [14].

b. Berg Balance Scale (BBS) [15].

The Berg Balance Scale (BBS) is a clinical tool designed to assess balance through the observation of 14 different functional tasks performed by the participant. The total score range is 0 (poor balance) to 56 points (excellent balance). It is a scale of 14 tests that quantitatively assesses the balance and risk of falling in elderly people by observing their performance. It needs 10 to 20 min to be completed and measures the patient’s ability to maintain balance either statically or by performing various functional movements for a specified period of time. The tests are scored from 0 to 4, with a score of 0 representing inability to complete and a score of 4 representing independent completion. The maximum score is 56. Scores from 0 to 20 represent impaired balance, 21 to 40 represent acceptable balance, and 41 to 46 represent good balance [16]. This scale measures both static and dynamic aspects of balance. It has good reliability (high inter-rater and intra-rater reliability) and validity (Cronbach α = 0.77; ICC = 0.95) [16]. It has been crossculturally validated in Greece by Lampropoulou et al. [15].

c. Timed Up-and-Go (TUG) [17].

This test assesses the mobility of elderly people [18]. Participants have to get up from a chair, walk three meters and back to it, and sit down as quickly as possible. Measurement is carried out with an electronic stopwatch and starts the moment the participant gets up from the chair and is completed when they sit down again. Normally, the time required to perform the test is 7–10 s [19]. The scale has good reliability and validity (Cronbach α = 0.98; ICC = 0.93) [20].

### 2.4. Procedures

Participants provided written, informed consent, and the study was performed according to the Declaration of Helsinki. The sample was taken from the region of Sparta, Greece, verifying that participants met the inclusion criteria. First, they were tested on the functional activities (TUG and FTSTS tests) and then the BBS. There was no training, but the procedure was explained to our subjects. Demographic characteristics were taken into consideration, such as age, smoking, number of falls, and other comorbidities.

### 2.5. Statistical Analysis

Descriptive statistics were applied to examine the demographic characteristics of the sample. Shapiro–Wilk test was used to examine normality. Spearman’s rank correlation coefficient (Spearman r) was used to explore the correlations between the demographic data, the functional activities, and the balance scale. The level of confidence was a = 0.05. Data were analyzed using IBM SPSS Statistics for Windows Version 29.0.1.0 (IBM Corp., Armonk, NY, USA).

## 3. Results

The demographic data are given in Table 1. Of the 35 participants, 45.7% (*n* = 16) were men and 54.3% were women (*n* = 19), and the mean age of the participants was 78 years (SD = 9.2). A total of 85.7% (*n* = 30) were not smoking, and 32 of the 35 participants had not experienced any falls. The comorbidities of the participants are detailed in Table 2. The mean performance on the Timed Up-and-Go (TUG) test was 15.1 s (SD = 6.4); on the Five Times Sit-to-Stand (FTSTS) test, it was 18.3 s (SD = 8.7); and on the Berg Balance Scale (BBS), it was 46.9 points (SD = 10.2) (Table 3).

According to our analysis, significant correlations were found between balance and functional performance variables, which means that people with better balance are more functional. Specifically, BBS scores were negatively correlated with TUG times (r = −0.83; *p* < 0.01) and FTSTS times (r = −0.69; *p* < 0.01), indicating that better balance was associated with faster performance in functional tests. Significant correlations were also observed among the three tests. The TUG and FTSTS tests were positively correlated (r = 0.76; *p* < 0.01), indicating that individuals who took longer to complete one test also performed slower on the other. Both the TUG and FTSTS tests were negatively correlated with BBS scores (r = −0.83 and r = −0.69, respectively; both *p* < 0.01), reflecting the association between better balance and better functional performance (Table 4).

Age was positively correlated with TUG (r = 0.66; *p* < 0.01) and FTSTS (r = 0.57; *p* < 0.01) scores, suggesting that older participants required more time to complete the tests because of the changes that occur as we grow older. A moderate negative correlation was also observed between age and BBS scores (r = −0.66; *p* < 0.01), indicating a decline in balance with increasing age. Regarding smoking status, current smokers demonstrated a negative correlation with item 14 of the BBS (r = −0.38; *p* < 0.05), suggesting reduced balance performance. Comorbidities were not associated with the functional tests, but a positive correlation was found between the comorbidities and performance on item 3 of the BBS (r = 0.34; *p* < 0.05) (Table 4).

The participants’ fall history was negatively correlated with performance on item 11 (turning 360 degrees) and item 14 (standing on one foot) of the Berg Balance Scale. Specifically, participants with a history of falls scored lower on item 11 (r = −0.36; *p* < 0.05) and item 14 (r = −0.41; *p* < 0.05), which may indicate that the falls are caused by a lack of balance (Table 4).

## 4. Discussion

The present study investigated the relationship between balance and functional mobility in a geriatric population, utilizing the Timed Up-and-Go (TUG), Five Times Sit-to-Stand (FTSTS), and Berg Balance Scale (BBS) assessments [21]. The findings revealed significant correlations among these tests, which may indicate that better balance is associated with improved functional performance. Specifically, higher BBS scores were linked to faster completion times in both the TUG and FTSTS tests.

These findings are consistent with prior studies reporting strong associations between balance and mobility tests in older populations. In particular, regarding the relationship between the Berg Balance Scale (BBS) and functional capacity in older adults, individuals with a total BBS score of less than 46 are more likely to have limitations in their daily living tasks according to Huang et al. [22]. However, the positive correlation between comorbidities and performance on item 3 of the BBS (which assesses the ability to maintain a standing position with eyes closed) was unexpected. One explanation could be that neurological conditions could affect postural control, making it difficult to balance with closed eyes. Additionally, musculoskeletal disorders like osteoarthritis may limit stability. Diabetes and hypertension both affect balance control negatively. These factors relating to reduced muscle strength, which are commonly observed in older adults, could explain the poor performance on this specific task of the BBS.

The findings of this study may indicate that age is associated with performance on the Timed Up-and-Go (TUG) test, with older individuals generally requiring more time to complete the task. This association reflects the natural declines in mobility, balance, and functional capacity that occur with aging. Aging leads to a progressive decline in both mental and physical capabilities. A gradual reduction in physical activity is a typical aspect of the aging process. One of the most significant consequences of aging is sarcopenia, which involves the loss of muscle mass, strength, and functional ability. Maintaining sufficient muscle strength, an efficient gait, and good balance—all of which tend to decline with age—is essential for preserving independence and overall well-being. Understanding how aging negatively affects balance and strength-related parameters is important for both healthcare professionals and older individuals [23]. These results are consistent with previous studies, which have similarly reported that increasing age is correlated with slower TUG performance [21]. Moreover, the FTSTS test is used as a screening tool for sarcopenia, so lower performance in older adults in the FTSTS test may be caused by decreased muscle mass [24].

The observed negative correlation between smoking status and performance on item 14 of the Berg Balance Scale (standing on one leg) may be attributed to the detrimental effects of smoking. This task requires significant postural control and lower limb strength. Among the assessment tools for balance and mobility assessment measures used in this study, only the BBS was significantly associated with the number of falls. The fall history was negatively correlated with performance on item 11 (turning 360 degrees) and item 14 (standing on one foot) of the Berg Balance Scale. Specifically, participants who reported previous falls had lower scores on item 11 and item 14. In another study based on a geriatric population with chronic stroke, it was shown that BBS scores may not differ significantly between people with and without a history of falls [12].

Specific BBS items, such as items 11 and 14, show stronger associations with variables like age, number of falls, and smoking status. Those tasks require postural control and lower limb strength, which tend to decline with age and are negatively affected by factors such as smoking or a history of falls. Therefore, this item may be more sensitive to age-related consequences in balance performance [25].

Around one out of three people over the age of 65 have a fall each year [26]. Common risk factors for falling include decreased muscle strength, slower reflexes, reduced proprioception, and diminished mobility, all of which are frequently associated with aging. Engaging in balance and flexibility exercises and strength and resistance training, as well as practices like tai chi, can enhance agility, coordination, and balance, helping to prevent or even reverse these age-related changes. Overall, regular physical activity lowers the incidence of falls over time and decreases the proportion of older adults who experience one or more falls [26].

There are other factors, such as BMI and use of medication, that were not included in our study and have been found to affect balance. In another study, both BMI and medication were found to significantly predict not only objective balance ability but also subjective balance confidence [27].

Based on the results of this study, incorporating multidimensional balance assessments into geriatric screening protocols may be helpful. Identifying impairments in specific BBS items may provide more targeted intervention strategies for fall prevention. Functional mobility tests such as TUG and FTSTS, in combination with balance scales, could be useful tools in clinical decision-making. The assessment tools used in this study—the Timed Up-and-Go (TUG) test, the Berg Balance Scale (BBS), and the Five Times Sit-to-Stand (FTSTS) test—are validated and reliable tools that are commonly used to assess functional mobility and balance among older adults. These tools have been extensively utilized in geriatric research and demonstrate high reliability and validity [14,16,20]. Their widespread use in similar studies supports their appropriateness for assessing functional performance and balance in community-dwelling older individuals, as in the present research [12,21,22,28]. However, in the present study, only the duration to complete each TUG task was noted, without taking into consideration other spatial and temporal parameters of gait, such as number of steps or stride length. While the TUG test is widely used in the clinical practice, it primarily reflects only one aspect of motor performance. Additionally, the small sample size limits the generalization of the findings to the entire elderly population.

Future studies should have larger samples with different ages. It is also important to examine differences between male and female elderly people and to investigate any gender differences in lower limb strength, dynamic balance, and balance confidence, as the susceptibility of older individuals to different types of tasks or to more challenging tasks has not been thoroughly explored. Also, further research with additional gait assessments will likely provide more information about older adults’ motor performance. Finally, it seems necessary to conduct a study with older people with a history of falls, not only to increase the sensitivity of the TUG test—for example by adding a cognitive load—but also to create new valid and reliable tests that can be easily applied in a clinical setting.

## 5. Conclusions

The results of this exploratory study may suggest an association between balance and functional status in the elderly population, indicating that impairments in one may be reflected in the other. Additionally, both balance and functional performance were significantly influenced by age and the presence of comorbidities. These findings focus on the importance of routine assessment of both parameters in older adults, as integrating balance and functional evaluations into geriatric care can support early detection of mobility limitations, help prevent falls, assist with independent living, and improve the overall quality of life in older adults. Future research with larger and more diverse samples is warranted. Stratifying results by gender, age groups, or baseline functional levels may further clarify these relationships and offer more comprehensive insights.

## Figures and Tables

**Table 1:** Demographic data of the sample (*n* = 35).

		Number	Min	Max	Mean	SD	Skewness	Kurtosis
Age			62	96	78	9.23		
Sex	Male	16			45.7%			
	Female	19			54.3%			
Number of Falls	No	32					4.20	18.09
	1	1						
	3	1						
	5	1						
Smoking status	Yes	5			14.3%		2.13	2.70
	No	30			85.7%			

**Table 2:** Sample’s comorbidities (*n* = 35).

Comorbidity	*n*
Hypertension	15
Polyarthritis	1
Pancreatitis	1
Diabetes	8
Glaucoma	1
Prostatic hyperplasia	1
Pituitary cancer	1
Thrombophilia	1
Osteoporosis	2
Vertigo	2
Hypothyroidism	6
Heart failure	1
Atrial fibrillation	4

**Table 3:** Descriptive statistics for the instruments.

	Min	Max	Mean	SD
Timed Up-and-Go	7.28	34.80	15.11	6.47
Five Times Sit-to-Stand	7.00	45.00	18.36	8.72
Berg Balance Scale	18.00	56.00	46.91	10.23

**Table 4:** Spearman’s r correlations between the demographic data, the functional tests, and the Berg Balance Scale.

	Timed Up-and-Go	Five Times Sit-to-Stand	Total Berg Balance Scale	BBS3	BBS14
Age	0.66 **	0.57 **	−0.66 **	−0.48 **	−0.49 **
Comorbidities	0.03	0.02	−0.04	0.34 *	0.02
Falls	0.19	0.19	−0.30	−0.10	−0.41 *
Smokers	0.11	−0.14	−0.22	−0.23	−0.38 *
Timed Up-and-Go	1.00	0.76 **	−0.83 **	−0.46 **	−0.70 **
Five Times Sit-to-Stand	0.76 **	1.00	−0.69 **	−0.46 **	−0.45 **
Total Berg Balance Scale	−0.83 **	−0.69 **	1.00	0.56 **	0.76 **

* *p* < 0.05 and ** *p* < 0.01.

## Data Availability

The data presented in this study are available upon request from the corresponding author. The data are not publicly available due to internal regulations.

## References

[1] Wróblewska Z., Chmielewski J.P., Florek-Łuszczki M., Nowak-Starz G., Wojciechowska M., Wróblewska I.M. (2023). Assessment of functional capacity of the elderly. Ann. Agric. Environ. Med..

[2] Fleming K.C., Evans J.M., Weber D.C., Chutka D.S. (1995). Practical functional assessment of elderly persons: A primary-care approach. Mayo Clin. Proc..

[3] Eckardt N. (2016). Lower-extremity resistance training on unstable surfaces improves proxies of muscle strength, power and balance in healthy older adults: A randomized control trial. BMC Geriatr..

[4] Zahedian-Nasab N., Jaberi A., Shirazi F., Kavousipor S. (2021). Effect of virtual reality exercises on balance and fall in elderly people with fall risk: A randomized controlled trial. BMC Geriatr..

[5] Gschwind Y.J., Kressig R.W., Lacroix A., Muehlbauer T., Pfenninger B., Granacher U. (2013). A best practice fall prevention exercise program to improve balance, strength/power, and psychosocial health in older adults: Study protocol for a randomized controlled trial. BMC Geriatr..

[6] Rosendahl E., Gustafson Y., Nordin E., Lundin-Olsson L., Nyberg L. (2008). A randomized controlled trial of fall prevention by a high-intensity functional exercise program for older people living in residential care facilities. Aging Clin. Exp. Res..

[7] Halvarsson A., Franzén E., Ståhle A. (2015). Balance training with multi-task exercises improves fall-related self-efficacy, gait, balance performance and physical function in older adults with osteoporosis: A randomized controlled trial. Clin. Rehabil. Tion.

[8] Bortoli C.G., Piovezan M.R., Piovesan E.J., Zonta M.B. (2015). Balance, falls and functionality among elderly persons with cognitive function impairment. Rev. Bras. Geriatr. E Gerontol..

[9] Chen B., Li M., Zhao H., Liao R., Lu J., Tu J., Wu J. (2023). Effect of Multicomponent Intervention on Functional Decline in Chinese Older Adults: A Multicenter Randomized Clinical Trial. J. Nutr. Health Aging.

[10] Lopes M.J., Escoval A., Pereira D.G., Pereira C.S., Carvalho C., Fonseca C. (2013). Evaluation of elderly persons functionality and care needs. Rev. Lat. Am. Enferm..

[11] Park B.S., Noh J.W., Kim M.Y., Lee L.K., Yang S.M., Lee W.D., Kim J. (2016). A comparative study of the effects of trunk exercise program in aquatic and land-based therapy on gait in hemiplegic stroke patients. J. Phys. Ther. Sci..

[12] Alenazi A.M., Alshehri M.M., Alothman S., Rucker J., Dunning K., D’Silva L.J., Kluding P.M. (2018). Functional Reach, Depression Scores, and Number of Medications Are Associated With Number of Falls in People with Chronic Stroke. PM R J. Inj. Funct. Rehabil..

[13] Csuka M., McCarty D.J. (1985). Simple method for measurement of lower extremity muscle strength. Am. J. Med..

[14] Muñoz-Bermejo L., Adsuar J.C., Mendoza-Muñoz M., Barrios-Fernández S., Garcia-Gordillo M.A., Pérez-Gómez J., Carlos-Vivas J. (2021). Test-Retest Reliability of Five Times Sit to Stand Test (FTSST) in Adults: A Systematic Review and Meta-Analysis. Biology.

[15] Lampropoulou S., Gizeli A., Kalivioti C., Billis E., Gedikoglou I., Nowicky A.V. (2016). Cross cultural adaptation of Berg Balance Scale in Greek for various balance impairments. Her. Sch. Open Access.

[16] Telenius E.W., Engedal K., Bergland A. (2015). Inter-rater reliability of the Berg Balance Scale, 30 s chair stand test and 6 m walking test, and construct validity of the Berg Balance Scale in nursing home residents with mild-to-moderate dementia. BMJ Open.

[17] Podsiadlo D., Richardson S. (1991). The timed “Up & Go”: A test of basic functional mobility for frail elderly persons. J. Am. Geriatr. Soc..

[18] Nicolini-Panisson R.D.A., Donadio M.V.F. (2013). Timed Up & Go test in children and adolescents. Rev. Paul. Pediatr..

[19] Joung H.J., Lee Y. (2019). Effect of creative dance on fitness, functional balance, and mobility control in the elderly. Gerontology.

[20] Hwang R., Morris N.R., Mandrusiak A., Mudge A., Suna J., Adsett J., Russell T. (2016). Timed Up and Go Test: A Reliable and Valid Test in Patients with Chronic Heart Failure. J. Card. Fail..

[21] Allison L.K., Painter J.A., Emory A., Whitehurst P., Raby A. (2013). Participation restriction, not fear of falling, predicts actual balance and mobility abilities in rural community-dwelling older adults. J. Geriatr. Phys. Ther..

[22] Wennie Huang W.N., Perera S., VanSwearingen J., Studenski S. (2010). Performance measures predict onset of activity of daily living difficulty in community-dwelling older adults. J. Am. Geriatr. Soc..

[23] Rezaei A., Bhat S.G., Cheng C.H., Pignolo R.J., Lu L., Kaufman K.R. (2024). Age-related changes in gait, balance, and strength parameters: A cross-sectional study. PLoS ONE.

[24] Park T.S., Shin M.J. (2024). Comprehensive Assessment of Lower Limb Function and Muscle Strength in Sarcopenia: Insights from the Sit-to-Stand Test. Ann. Geriatr. Med. Res..

[25] Wang C.Y., Hsieh C.L., Olson S.L., Wang C.H., Sheu C.F., Liang C.C. (2006). Psychometric properties of the Berg Balance Scale in a community-dwelling elderly resident population in Taiwan. J. Formos. Med. Assoc. (Taiwan Yi Zhi).

[26] Eckstrom E., Neukam S., Kalin L., Wright J. (2020). Physical Activity and Healthy Aging. Clin. Geriatr. Med..

[27] Anson E., Thompson E., Odle B.L., Jeka J., Walls Z.F., Panus P.C. (2018). Influences of Age, Obesity, and Adverse Drug Effects on Balance and Mobility Testing Scores in Ambulatory Older Adults. J. Geriatr. Phys. Ther..

[28] Aartolahti E., Häkkinen A., Lönnroos E., Kautiainen H., Sulkava R., Hartikainen S. (2013). Relationship between functional vision and balance and mobility performance in community-dwelling older adults. Aging Clin. Exp. Res..

